# Therapeutic challenges in hepatitis C-infected injection drug using patients

**DOI:** 10.1186/1477-7517-3-31

**Published:** 2006-11-10

**Authors:** Curtis L Cooper, Edward J Mills

**Affiliations:** 1 Associate Professor of Medicine-University of Ottawa Hospital, Division of Infectious Diseases-The Ottawa Hospital, Ottawa, Canada; 2 Centre for International Health and Human Rights Studies, Toronto, Canada; 3 Faculty of Health Sciences, Clinical Epidemiology and Biostatistics, McMaster University, Hamilton, Canada

## Abstract

Hepatitis C Viral (HCV) infection in the injection drug user (IDU) population is a major medical concern. Concurrent substance abuse, co-morbid mental health conditions, poor socioeconomic status and a complex treatment protocol that is often incompatible with the life styles of IDUs combine to account for poor uptake and completion of HCV treatment. This article discusses HCV antiviral treatment issues relevant to IDUs chronically infected with this virus. The effect of non-injected substances of abuse on treatment outcome is considered. Priority issues requiring research are discussed.

## Background

Given its high incidence and prevalence, the complications of chronic hepatitis C virus (HCV) infection will impact the morbidity and mortality of at-risk populations in the developed world for the foreseeable future [1]. Those with substance abuse concerns, and particularly injection drug users (IDUs), are at a greater risk of HCV infection [2]. In North America, injection drug use remains the primary risk factor for new and chronic infections. In Canada, injection drug use is estimated to account for over half of all current HCV infections [3]. Approximately 55% of active and 49% of post injection drug users are HCV infected [3]. Although therapy is available for HCV infection, there are multiple obstacles that diminish the likelihood of past and present IDUs receiving combination interferon and ribavirin HCV treatment. In this article, we discuss relevant HCV antiviral treatment issues pertaining to IDUs chronically infected with HCV. We additionally consider the effect of other substances of abuse on treatment and priority issues requiring research.

### Influence of substance use on natural history of HCV

Not all individuals with chronic HCV infection will progress to advanced stages of fibrosis and end-stage liver disease. During the initial period of evaluation of a HCV infected patient, the physician formulates an impression regarding the likelihood of poor outcome based on risk factors for rapid progression (e.g. immune suppression, alcohol use), physical examination, laboratory evaluation and liver biopsy. Although the final decision to initiate HCV antiviral therapy is made on a case-by-case basis, treatment is strongly recommended for those having or at least predicted to have more progressive disease.

The practice of drug injection is not known to directly influence the rate of HCV progression despite the potential for multiple repeat exposures to various genotypes and quasi-species. However, there are several factors associated with injection drug use which may contribute to accelerated progression [4]. Chief among these is excess alcohol use which is generally defined as more that 50 grams of alcohol per day (i.e. approximately 3–4 beers per day). Concurrent excess alcohol consumption is found in 40% of active IDUs [5]. In our own clinic, we found that 75% of past injection drug users have a history of excess alcohol use compared to 32% in those who acquired HCV infection through other means (χ^2^, p < 0.001). In addition to increasing risk behaviors [6], alcohol is well known to accelerate the rate of hepatic fibrosis and reduce the time to cirrhosis in HCV [7-9]. Clearly, concurrent alcohol use in HCV is a factor predicting increased need for HCV therapy but is also a factor limiting eligibility for access to therapy. Furthermore, HCV antiviral therapy efficacy is diminished in those engaging in excess alcohol use (see below). As a primary measure, support for alcohol reduction and cessation is needed in those with HCV, irrespective of injection drug use.

Although not supported by strong evidence, it is plausible that poor nutrition may play a role in influencing HCV progression. Relative malnutrition and micronutrient deficiency likely compromise the livers ability to control chronic infection, contain inflammation and resorb fibrotic material as it is produced [10]. Nutrition is often substandard in substance users. Deficient food intake, anorexia, nausea, vomiting, poor gastrointestinal absorption, altered metabolism and increased energetic expenditure contribute to a poor nutritional status in alcoholics [11,12] and injection drug users [13].

### Factors influencing treatment consideration

#### Psychiatric health

Psychiatric health is an important variable to consider when determining the initiation of HCV antiviral therapy. Those living with chronic HCV infection and those with current or past injection drug use [14] have a heavy burden of psychiatric illness [15-18]. In our clinic, depression was identified in 62% of patients at initial evaluation using the Center for Epidemiologic Studies of Depression Scale (CES-D) [18,19]. To compound this problem, interferon-based HCV therapies are well-known to increase the frequency and severity of depression [20] and other psychiatric illness [16]. The high frequency of depression among HCV-infected patients is multifactoral. As in other populations with chronic illnesses, such as cancer and diabetes, alcohol intake, smoking, and depression often co-occur and influence mental well-being [21-24]. The inter-relationships between the bio-psycho-social variables within HCV-infected injection drug users are not fully understood and deserve continued study. We and other groups are evaluating the use of prophylactic antidepressants in those initiating interferon-based HCV treatment [25,26].

#### Socioeconomic challenges

There are several concrete obstacles to the successful delivery of HCV antiviral therapy to IDUs. Suitable shelter is not always available and a lack of safe storage facilities for HCV therapies negates successful adherence and treatment success. The absence of refrigeration, which is required for interferon storage between weekly dosing, may compromise patients' ability to receive HCV therapy. Poor attendance for outpatient clinical assessments is partially explained by limited or cost-prohibitive transportation options.

Other obstacles to treatment are less tangible. Mistrust of the medical community may influence patient willingness to be assessed and initiation of HCV antiviral therapy. This is, in part, fostered by multiple sources of inaccurate or misinterpreted information pertaining to HCV disease and treatment that can be found in the lay literature and on the internet. Another contributing factor is the poor treatment that many IDUs have experienced from law enforcement services and medical personnel. The negative view of IDUs among many in the medical community [27] necessitates that medical personnel consider the complex socioeconomic obstacles that placed the patient in the risk category to begin with. This mistrust represents an additional obstacle to the diagnosis and delivery of health care, including HCV antiviral therapy. Recent guidelines and commentaries have clearly stated that ongoing substance abuse should not preclude consideration for treatment [28,29]. This attitude has resonated within the community of health care professionals delivering HCV care, which seem more willing to consider treatment in this population, assuming that a reasonable degree of stability in substance use has been achieved by the patient.

A further concern for physicians dealing with HCV IDU patients is the patient's exposure to physical and sexual violence [30]. Populations at risk for physical and sexual violence are at a heightened risk for exposure to HIV and viral hepatitis infections. Although intervening on behalf of patients by physicians is challenging, we should be aware that this population is at an increased risk for violence and that initial intake discussions should specifically target issues of physical and sexual violence.

### Therapeutic outcome

#### Influence of injection drug use

For patients no longer using injection drugs, the success of therapy is determined by the same predictors (e.g. adherence, genotype) as those without a history of injection drug use. For those with active use, issues related to adherence, tolerance and effectiveness, psychological health, and risk of re-infection are important considerations.

In general, adherence to antimicrobial therapy is diminished in active drug users [31-33]. However, studies demonstrate that adherence varies widely among IDU patients and can, in some cases, approach that of populations without substance abuse [34-36]. Programs with special expertise in providing care for drug users can increase adherence rates to as high as 80% [37-42].

There is little conclusive data to suggest that HCV antiviral therapy is tolerated less well or is less effective in those with concurrent injection use. In fact, in one study, sustained virological response rates comparable to other HCV-treated populations were achieved despite relapses to drug use in 80% of the patients receiving therapy [43]. This group attributed their success to providing multidisciplinary care that included expertise in liver disease and substance abuse management. Other studies replicate these findings. A 29% sustained virological response rate was achieved in a population of 66 methadone recipients [44]. This cohort had a high level of concurrent psychiatric illness (80%), concurrent alcohol use while on therapy (20%) and one-third relapsed into illicit drugs use. Therapy was effectively and safely delivered by provision of mental health care and appropriate resources to stabilize ongoing substance use.

Re-infection is often cited as justification to withhold HCV antiviral treatment from those with ongoing injection drug use. Although there is a risk and documented examples of have been cited [45], re-infection is a rare occurrence. HCV is generally acquired early in the career of an IDU as a result of inexperience and lack of knowledge regarding safe injecting techniques. Most patients presenting to clinic for therapy have a long history of injection drug use and are therefore less likely to commit the same errors in safe needle use as their junior counterparts. Of course, education and support for safe injection practices is an important component of care delivery to those treated for HCV infection. Support for safe injection facilities, needle exchange programs and provision of injection equipment have been demonstrated to reduce infection rates [46-49]. It is reasonable to assume that this benefit would extend to those who successfully clear HCV with antiviral therapy.

#### Influence of alcohol on HCV treatment

Alcohol consumption impairs the efficacy of interferon-based HCV therapy [50,51]. There are several ways in which alcohol is thought to produce this negative influence on treatment outcomes. Most studies suggest that excess alcohol consumption increases HCV RNA levels [52-57]. In mice, alcohol consumption blunts HCV-specific T-helper and cytotoxic T lymphocytes response as well as cytokine expression [58,59]. Furthermore, decreased interferon-γ levels, resulting from alcohol-induced dendritic cell dysfunction [59,60] likely influence HCV RNA levels. Both HCV RNA level and immune status predict sustained virologic response rates to interferon-based HCV treatment.

Although ongoing controlled alcohol (or other substance abuse) should not preclude HCV drug therapy [46], cessation of alcohol use should be emphasized as a highly beneficial therapeutic intervention. Physicians are in an optimal situation to encourage alcohol reduction strategies and brief discussions with patients may reduce alcohol intake [61]. With successful alcohol reduction, liver inflammation and fibrosis will be reduced, HCV RNA levels will decline, and the probability of response to HCV drug therapy will be increased. This intervention requires sustained patient and physician commitment to alcohol cessation programs and patience.

### Other substances of concern

#### Influence of smoking and marijuana use

High rates of cigarette smoking have been reported among HCV patients in Canada, Europe, and the Far East[18,62-64]. In our own cohort, the rate of cigarette smoking (63%) reported in HCV infection was much higher than the Canadian national average rate of 24% [18,65]. Smoking is also common among injection drug users [66].

Cigarette smoking has many known negative health consequences [67] and has been consistently associated with reduced health-related quality of life [18,67,68]. The negative physical effects of smoking have been found to be more severe in those with chronic medical conditions, including liver disease. In a cross-sectional study of 6095 individuals with HCV, cigarette smoking along with alcohol abuse were both independently associated with elevated ALT levels [69]. Tobacco smoking has also been linked to hepatocellular carcinoma [21,22] and increased Knodell fibrosis [63]. Among Japanese patients with chronic liver disease (67% were HCV positive), hepatocellular carcinoma was higher for smokers and was independent of alcohol use (Relative Risk 15.4) [22]. Smoking may also increase histological activity in chronic HCV patients irrespective of alcohol use [70]. These findings justify further systematic assessment of the impact of smoking on those living with chronic liver disease.

Cigarette smoking has been associated with clinically relevant decrements in physical and mental health-related quality of life in those chronically infected with HCV [18,71]. Given that cigarette smoking is a modifiable health behavior, these findings may have important clinical implications. Smoking cessation interventions may help improve health-related quality of life for those with HCV-infection [72,73]. Further study of the effectiveness of psychological and/or pharmacological treatments for smoking cessation and relapse prevention, and the impact of these interventions on HCV patients' long-term health-related quality of life are justified.

The tar in marijuana contains similar carcinogens to those found in tobacco cigarettes [74]. Furthermore, marijuana smoking has been associated with increased risk of head and neck cancer [74]. To be fair, patient testimony suggests that marijuana many play a valuable role in attenuating the many side effects associated with interferon and ribavirin-based HCV treatment. In fact, treatment outcomes have been reported to be improved in those using marijuana compared to those not [44]. Future research on the influence of marijuana smoking on liver disease progression, HCV health-related quality of life and HCV treatment efficacy should be pursued.

### Influence of HIV co-infection on HCV progression and treatment

As a result of common risk factors for exposure, HCV and HIV are often found concurrently. The pace of HCV-induced hepatic fibrosis is accelerated in those with HIV co-infection [7,75]. As a result, the burden of end stage liver disease and liver-related death is increased [76,77]. For these reasons, HCV treatment evaluation is essential in those with HIV-HCV co-infection. The likelihood of successfully clearing HCV infection with interferon and ribavirin-based therapy is diminished in those with HIV co-infection, even with well managed HIV disease [78,79]. Treatment side effects are not inconsequential but, in general, not substantially more plentiful or severe in co-infection [78,79]. Traditionally, there have been concerns pertaining to interactions between ribavirin and HIV antiretrovirals [80,81]. As didanosine, stavudine and zidovudine use diminish as safer alternates become available, concerns about nucleoside drug interactions with ribavirin have diminished. It is clear that pegylated interferon use in this immune compromised population does not result in a greater risk of infectious complications or HIV-defining opportunistic infections [78,82].

### Research priorities

Investigation and validation of treatment strategies which will improve adherence to viral hepatitis drug therapy are required to ensure that individuals who use injection drugs or other substances of abuse derive the maximal possible benefits from current viral hepatitis therapies. Given the wide spread use of medicinal marijuana in this population, quantification of the benefits and negative consequences of regular, long term use is mandatory. Rigorous evaluation of the safety, purported benefits and effect on quality of life are required for the many alternative and herbal remedies used by those living with HCV.

Many patients seek alternative sources of health care for a variety of reasons, including cultural relations, belief-systems, the concept of detoxifying their liver, and the potential therapeutic benefits of alternative therapies. Acupuncture, a traditional Asian intervention, often used for addiction and substance cravings, has been evaluated in a number of randomized trials. The evidence, has however, shown consistently that acupuncture is ineffective in addictive drug use [83,84]. A popular herbal medicine used by patients with liver disorders, including HCV, is Milk Thistle (Silybum marianum). Although the safety of Milk Thistle is now largely established [85], the therapeutic effectiveness is still uncertain. Some evidence suggests that it has therapeutic effectiveness in hepatitis B/C in reducing liver-related mortality. However, better quality studies indicate this result is uncertain [86]. There are currently large trials enrolling HCV patients to determine Milk Thistle's impact on HCV specific outcomes.

It is plausible that the course and outcome of HCV many differ in those infected by injection drug use. Inoculum size and frequency of exposure may perturb the viral-host immune interaction. The poor nutrition status of injection drug uses may influence HCV pathogenesis and response to therapy. Well-designed analyses of this issue, which control for key confounders including alcohol use and nutritional status would provide clarity.

The need to implement interventions to reduce the infection rate of HCV among IDUs is critical. Although ongoing research is vital to ensure optimal application of techniques to limit the spread of HCV in this high risk group, this should not be used as an excuse to delay introduction of practices which are now well known to be effective) [46-49].

## Conclusion

HCV infection in the IDU population is a major medical concern, one that is likely to remain for the foreseeable future. The difficulties of delivering current interferon-based HCV treatment to this population are well known. Co-morbid mental health concerns, concurrent substance use and abuse, poor socioeconomic status, exposure to violence and a treatment protocol that is often not compatible with the schedules of injection drug users combine to account for poor uptake and completion of HCV treatment. Moral and financial support from government for this disenfranchised population would perhaps provide the greatest impetus to allow for successful delivery of HCV treatment to this population. In a more favorable climate, practices demonstrated to reduce harmful behavior and effectively manage the above risk factors and co-morbidities in the IDU population could produce improvement in individual and population health. This, in turn would make HCV drug treatment more accessible to a larger proportion of those requiring therapy.
